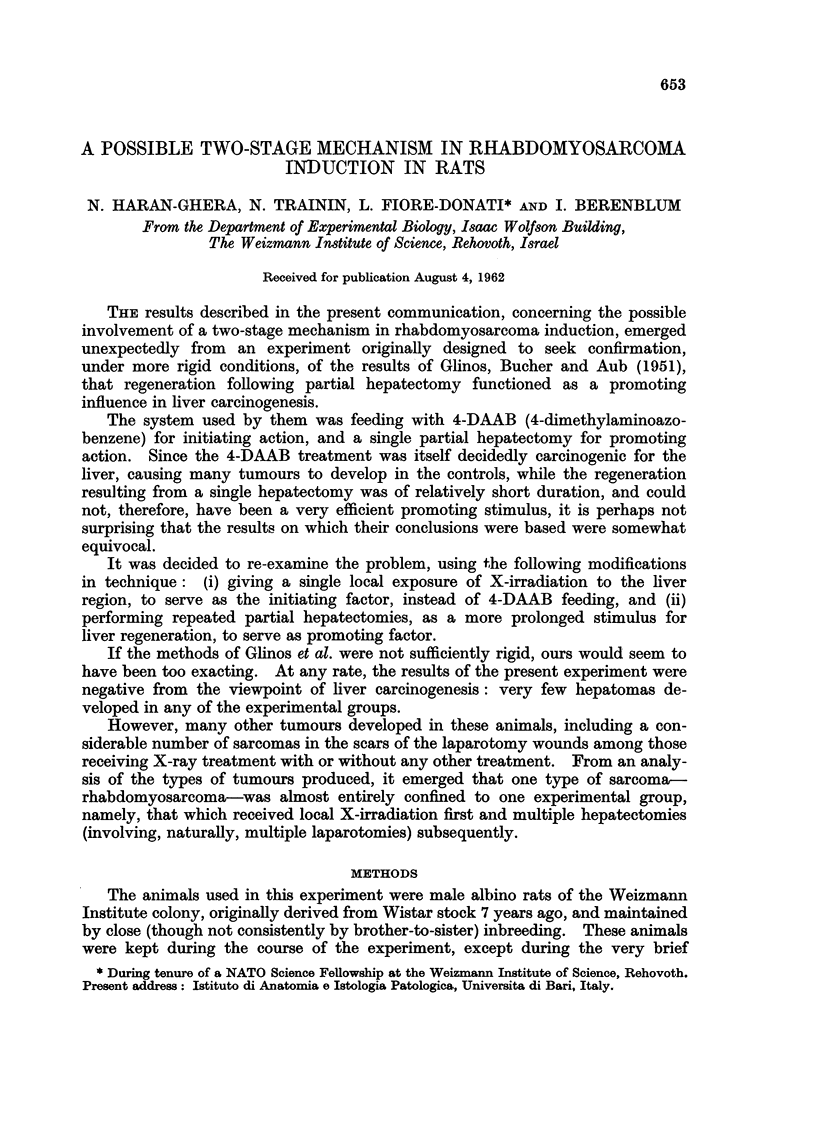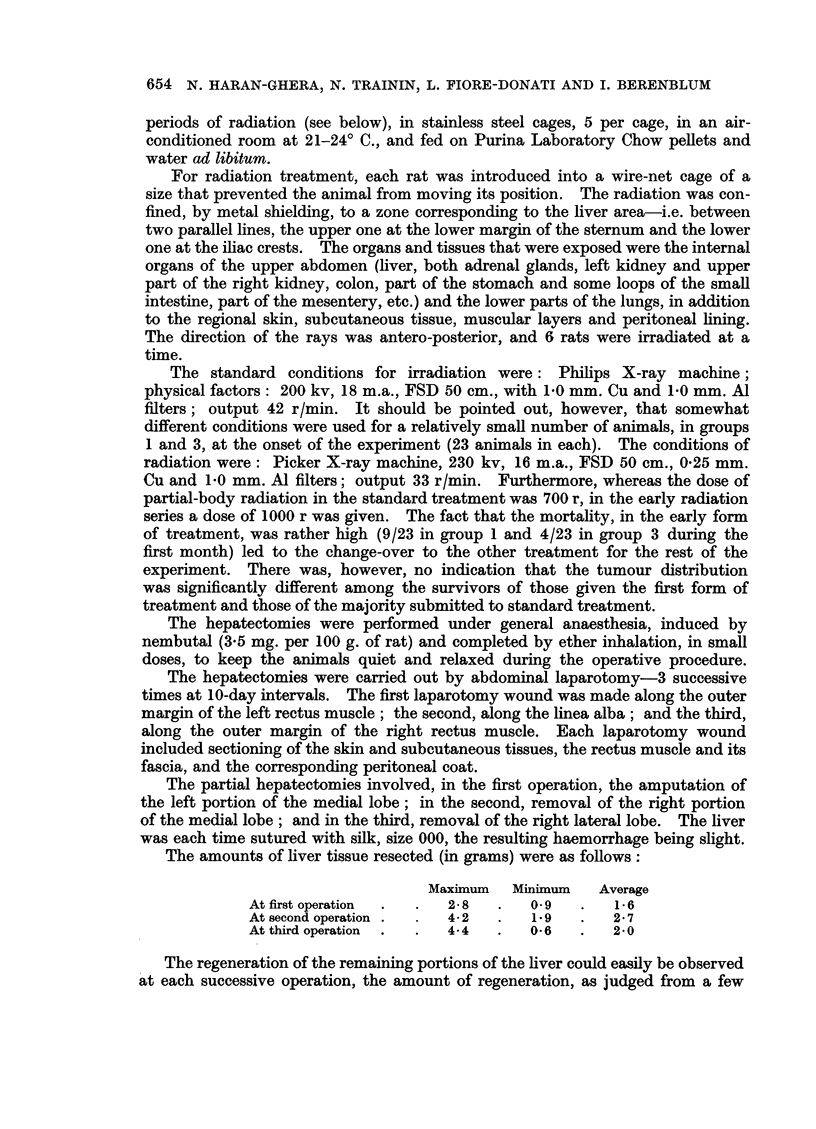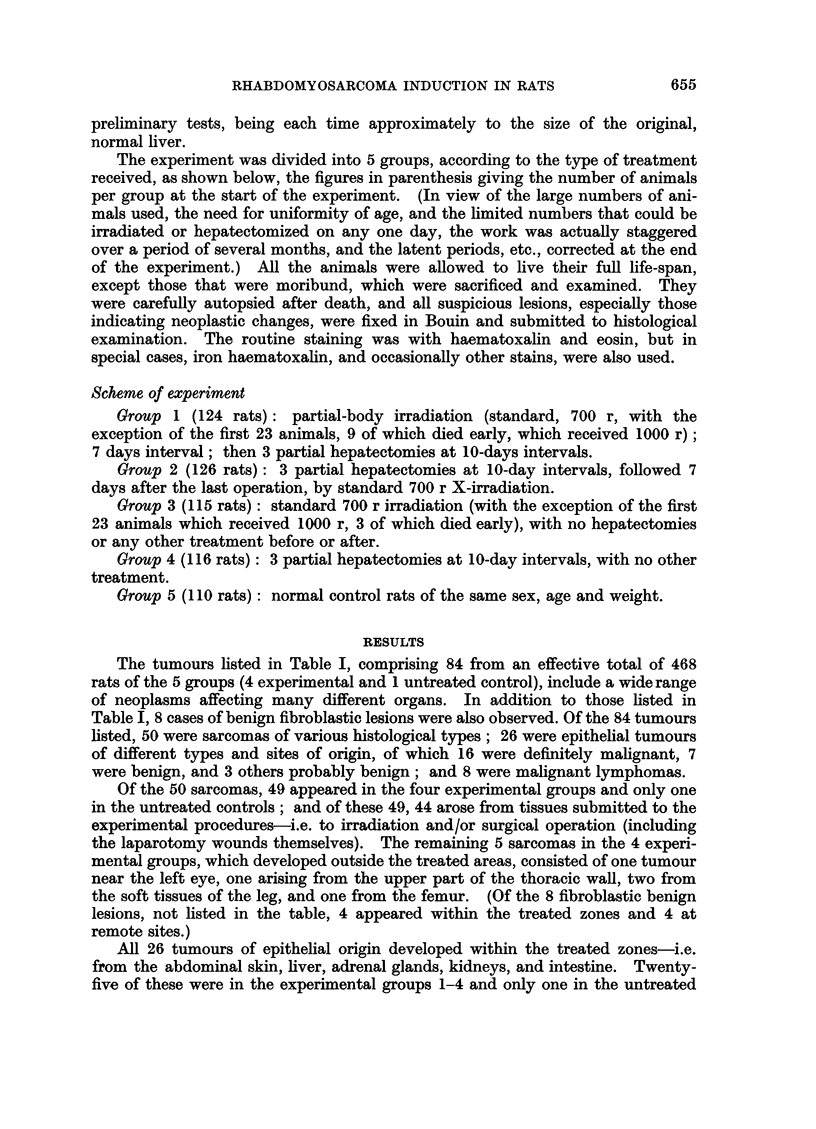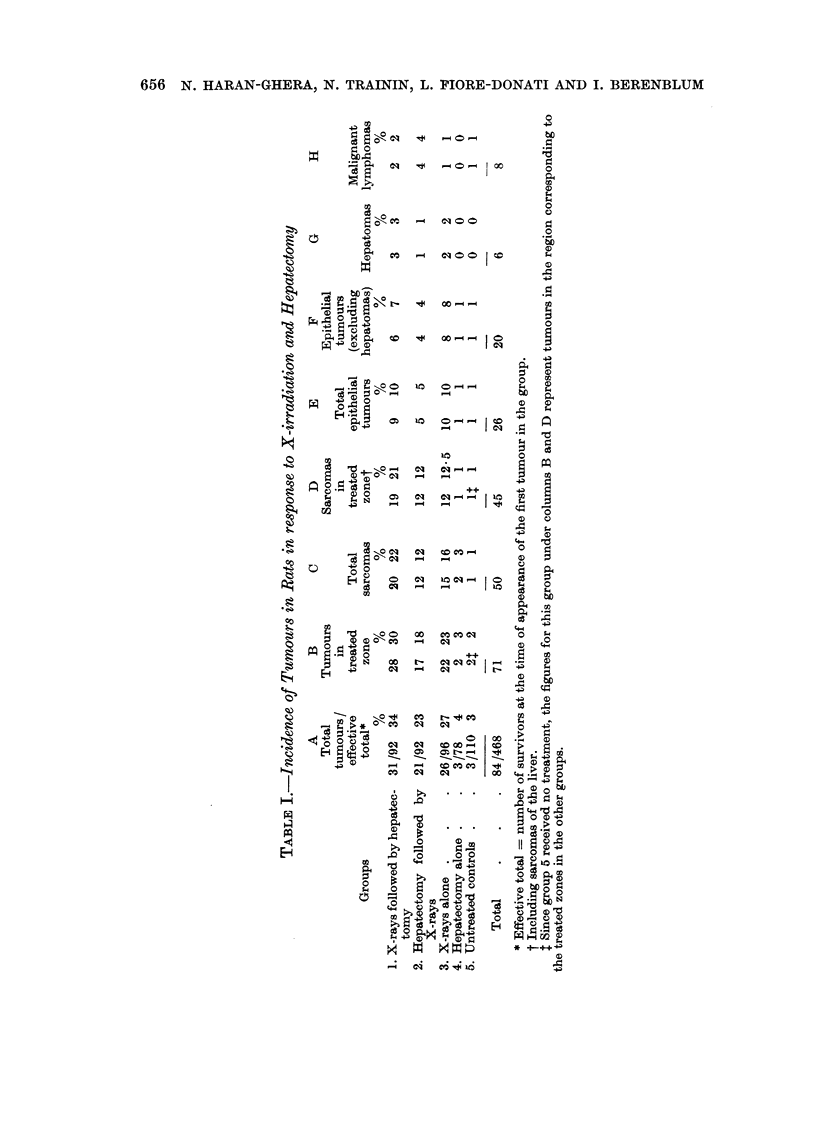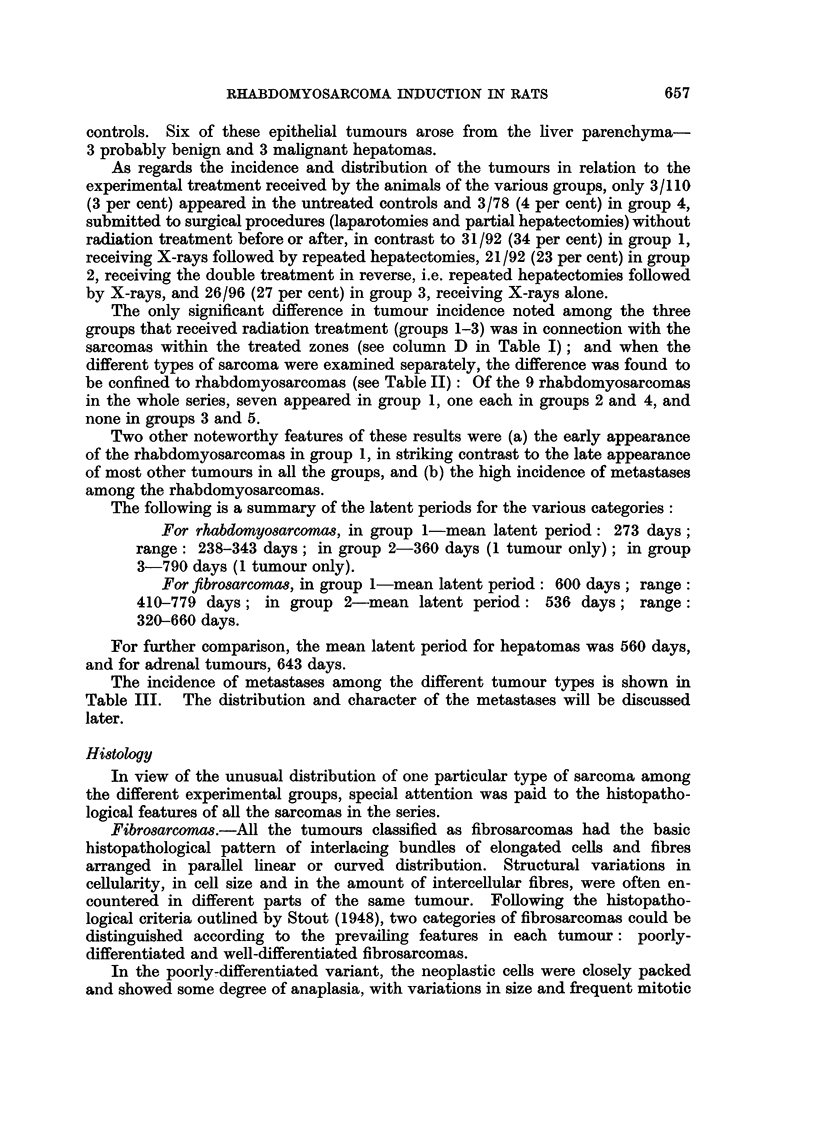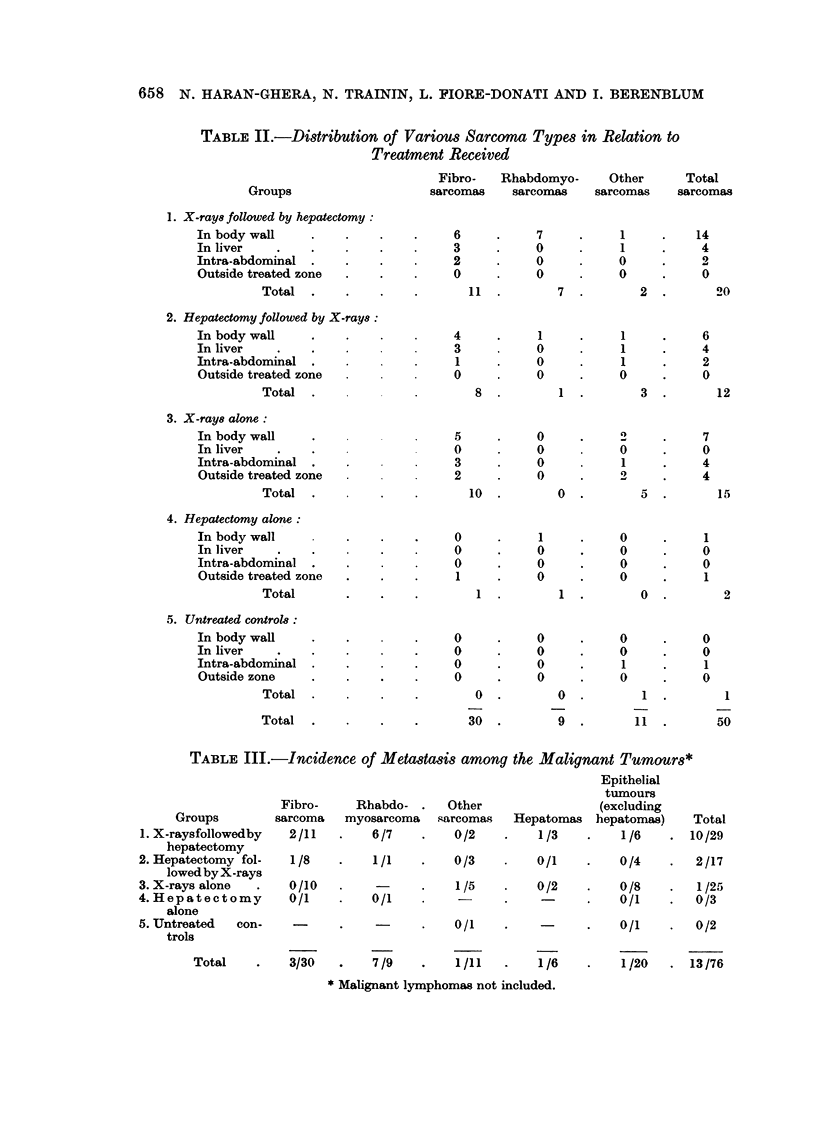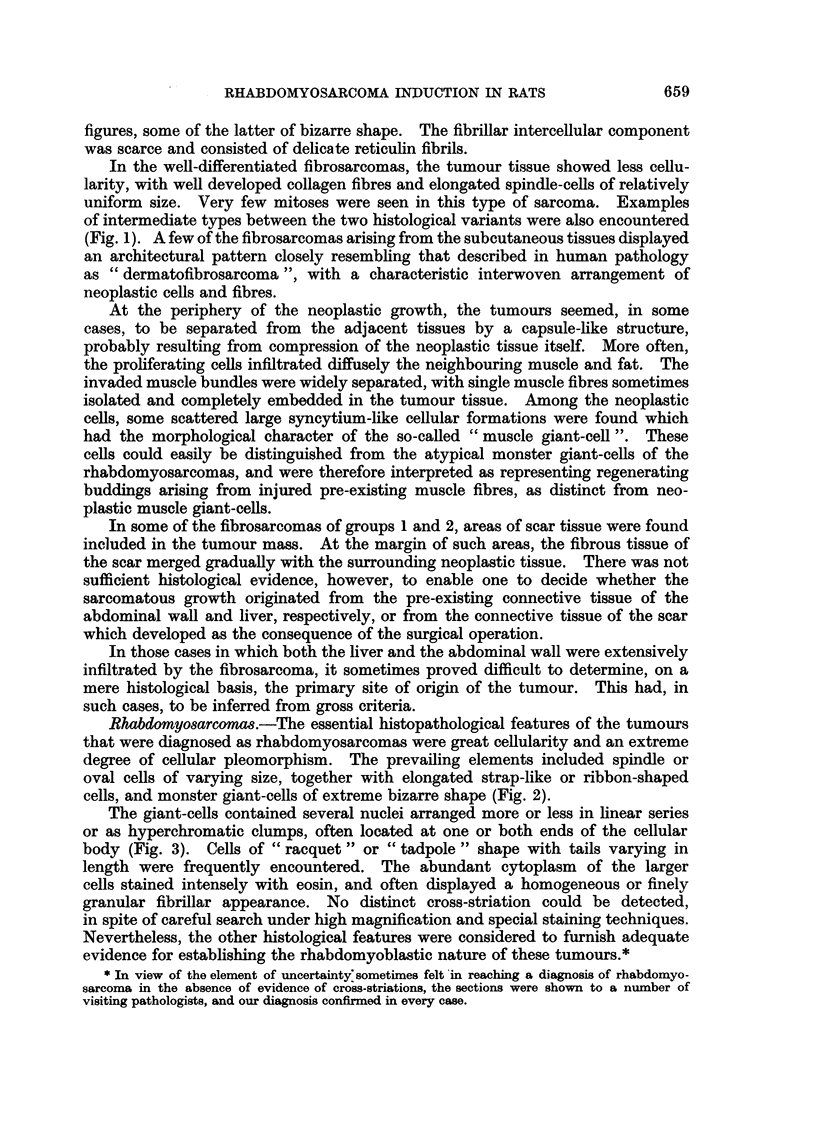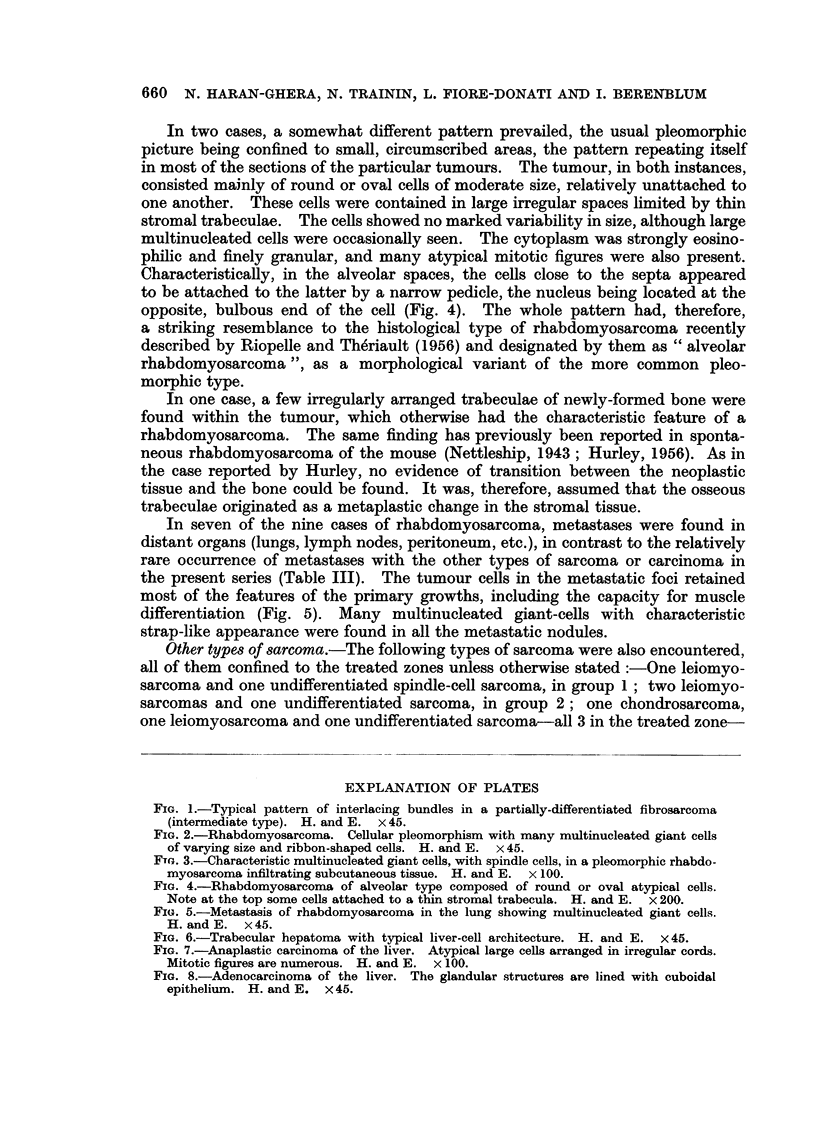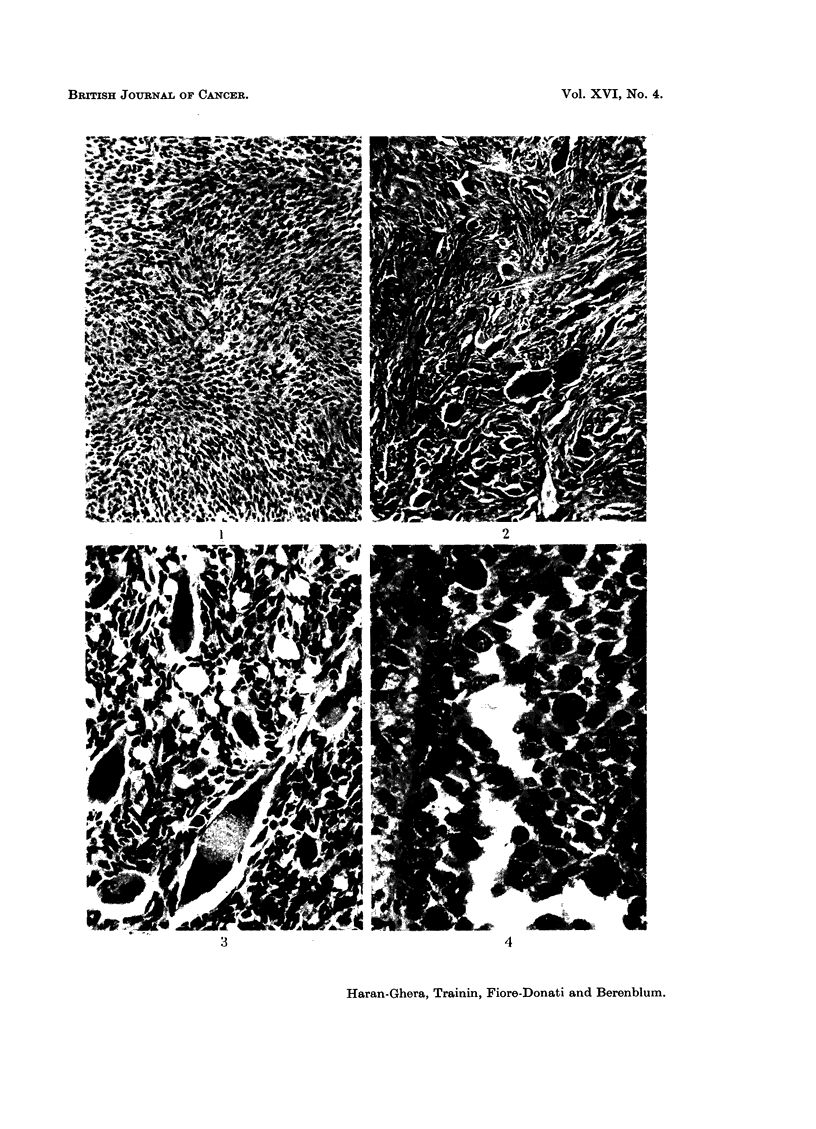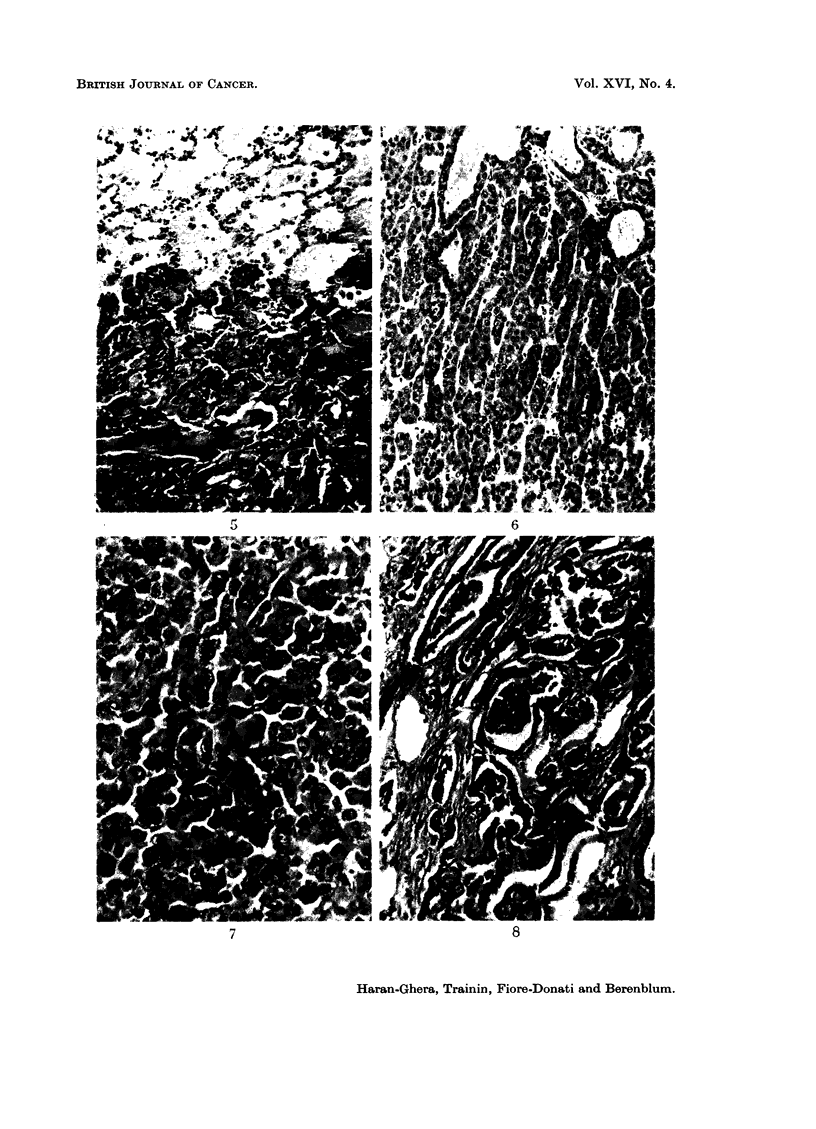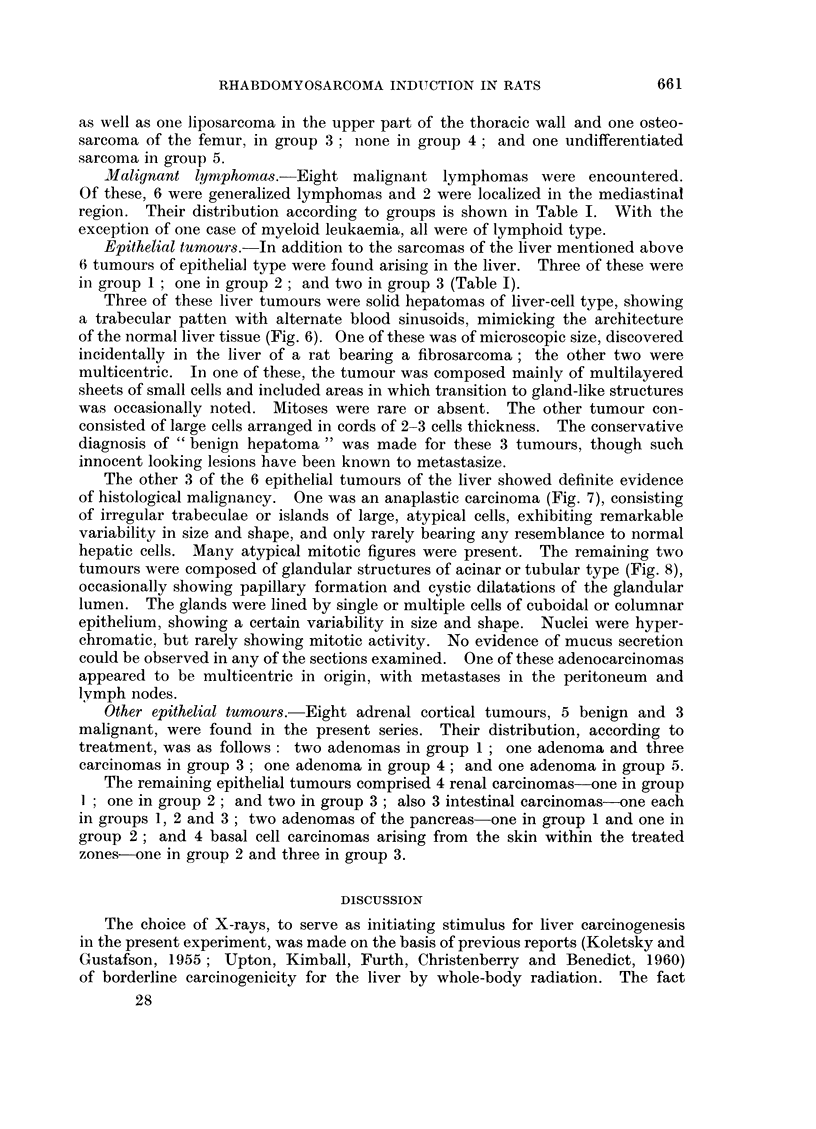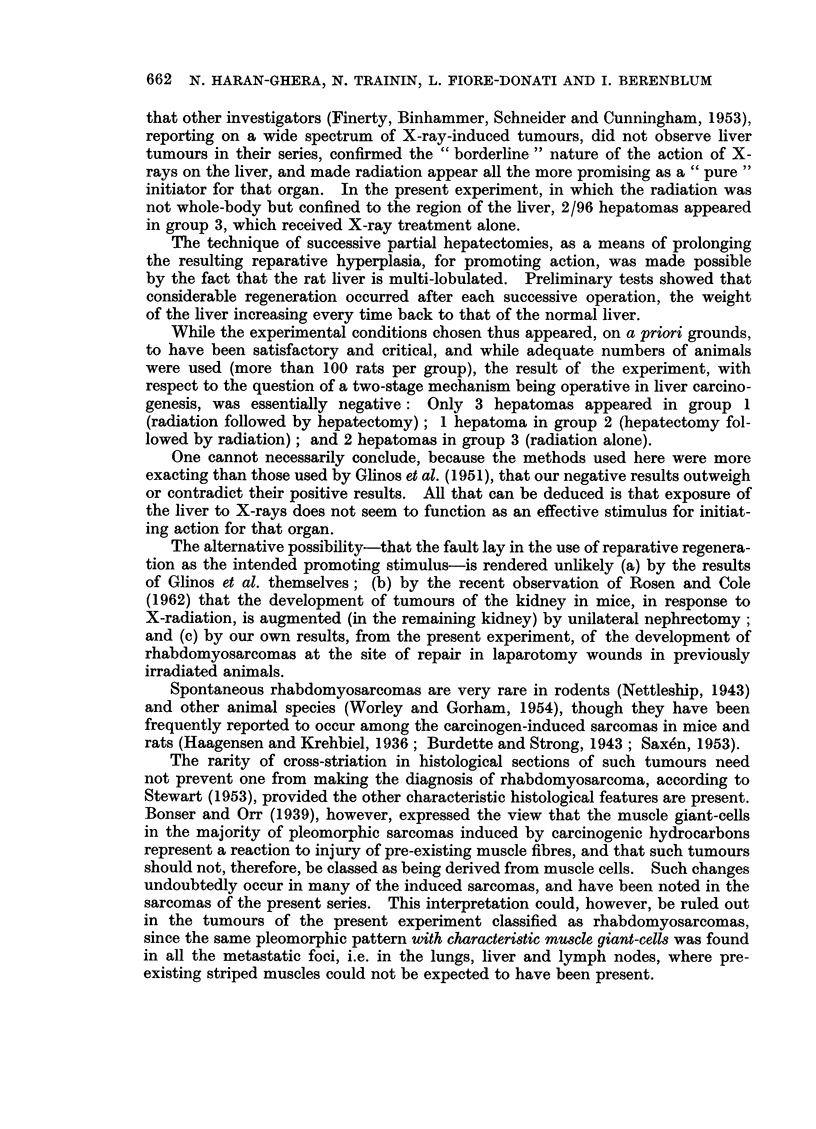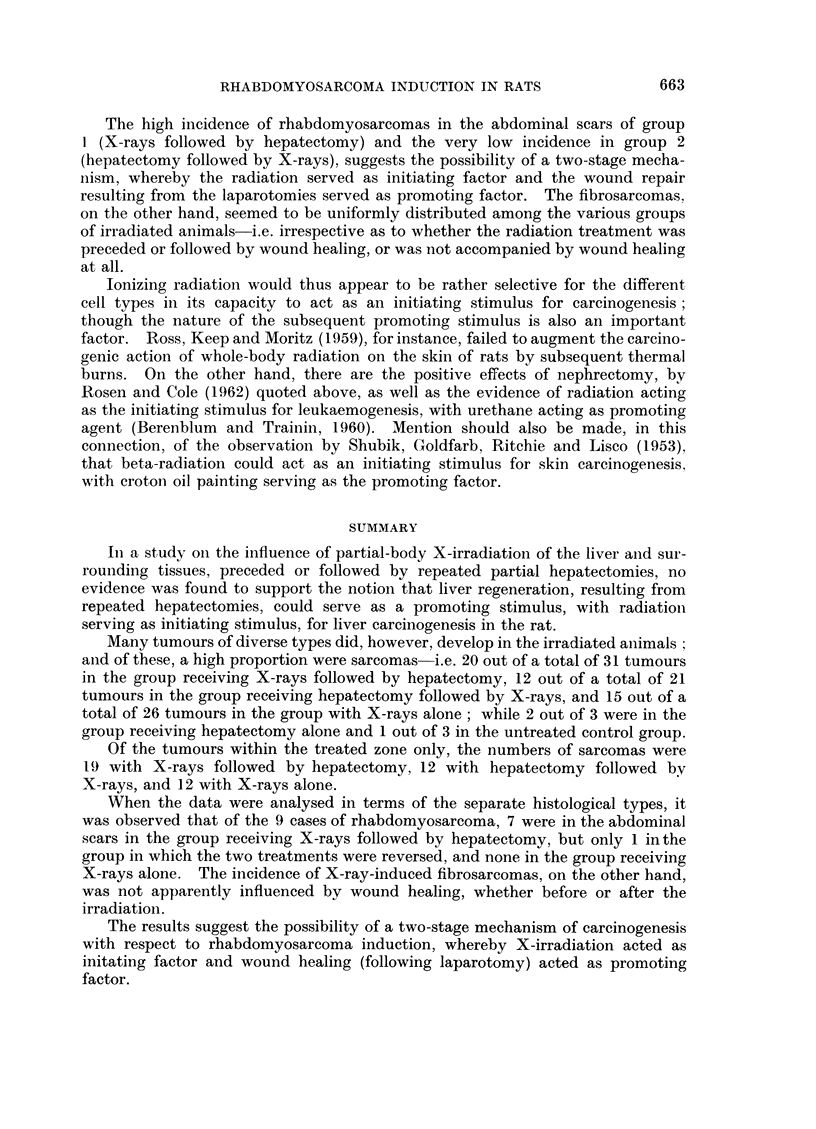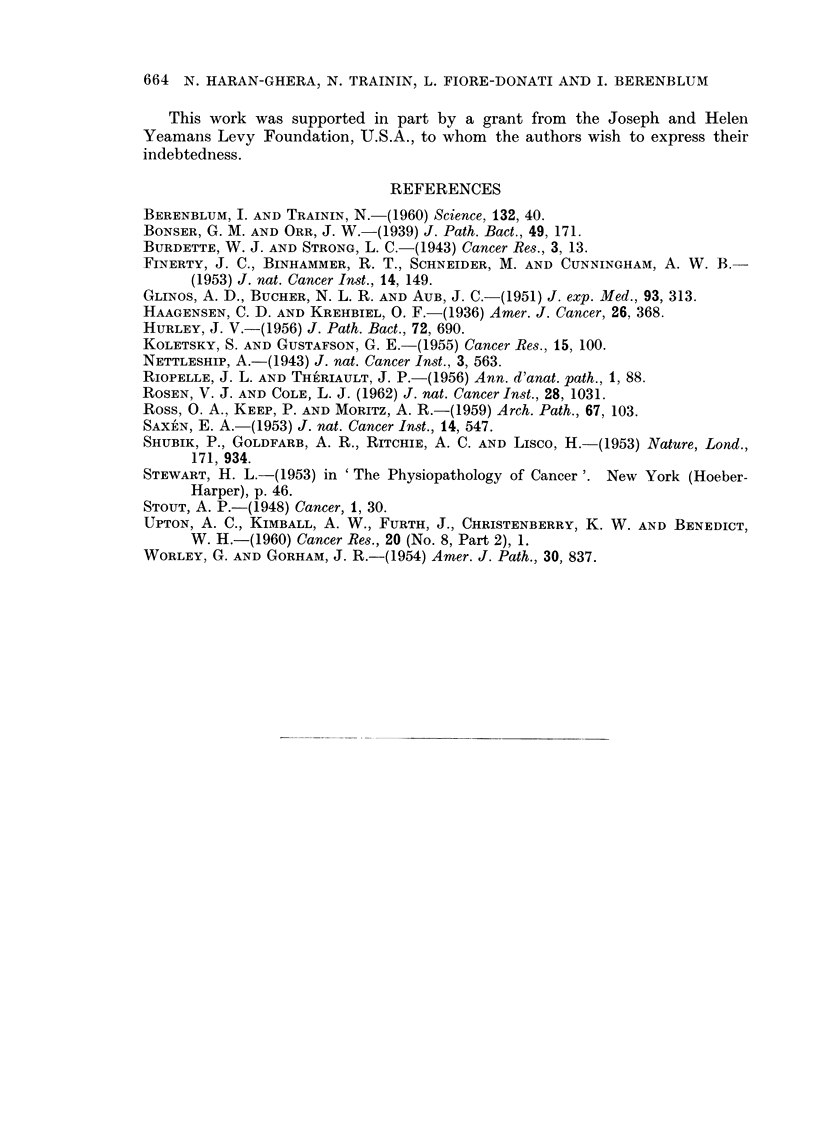# A Possible Two-Stage Mechanism in Rhabdomyosarcoma Induction in Rats

**DOI:** 10.1038/bjc.1962.76

**Published:** 1962-12

**Authors:** N. Haran-Ghera, N. Trainin, L. Fiore-Donati, I. Berenblum

## Abstract

**Images:**


### A POSSIBLE TWO-STAGE MECHANISM IN RHABDOMYOSARCOMA INDUCTION IN RATS

N. HARAN-GHERA, N. TRAININ, L. FIORE-DONATI* AND I. BERENBLUM

*From the Department of Experimental Biology, Isaac Wolfson Building, The Weizmann Institute of Science, Rehovoth, Israel*

Received for publication August 4, 1962

THE results described in the present communication, concerning the possible involvement of a two-stage mechanism in rhabdomyosarcoma induction, emerged unexpectedly from an experiment originally designed to seek confirmation, under more rigid conditions, of the results of Glinos, Bucher and Aub (1951), that regeneration following partial hepatectomy functioned as a promoting influence in liver carcinogenesis.

The system used by them was feeding with 4-DAAB (4-dimethylaminoazobenzene) for initiating action, and a single partial hepatectomy for promoting action. Since the 4-DAAB treatment was itself decidedly carcinogenic for the liver, causing many tumours to develop in the controls, while the regeneration resulting from a single hepatectomy was of relatively short duration, and could not, therefore, have been a very efficient promoting stimulus, it is perhaps not surprising that the results on which their conclusions were based were somewhat equivocal.

It was decided to re-examine the problem, using the following modifications in technique : (i) giving a single local exposure of X-irradiation to the liver region, to serve as the initiating factor, instead of 4-DAAB feeding, and (ii) performing repeated partial hepatectomies, as a more prolonged stimulus for liver regeneration, to serve as promoting factor.

If the methods of Glinos *et al.* were not sufficiently rigid, ours would seem to have been too exacting. At any rate, the results of the present experiment were negative from the viewpoint of liver carcinogenesis : very few hepatomas developed in any of the experimental groups.

However, many other tumours developed in these animals, including a considerable number of sarcomas in the scars of the laparotomy wounds among those receiving X-ray treatment with or without any other treatment. From an analysis of the types of tumours produced, it emerged that one type of sarcoma—rhabdomyosarcoma—was almost entirely confined to one experimental group, namely, that which received local X-irradiation first and multiple hepatectomies (involving, naturally, multiple laparotomies) subsequently.

### METHODS

The animals used in this experiment were male albino rats of the Weizmann Institute colony, originally derived from Wistar stock 7 years ago, and maintained by close (though not consistently by brother-to-sister) inbreeding. These animals were kept during the course of the experiment, except during the very brief

* During tenure of a NATO Science Fellowship at the Weizmann Institute of Science, Rehovoth. Present address : Istituto di Anatomia e Istologia Patologica, Universita di Bari, Italy.

periods of radiation (see below), in stainless steel cages, 5 per cage, in an air-conditioned room at 21–24° C., and fed on Purina Laboratory Chow pellets and water *ad libitum.*

For radiation treatment, each rat was introduced into a wire-net cage of a size that prevented the animal from moving its position. The radiation was confined, by metal shielding, to a zone corresponding to the liver area—i.e. between two parallel lines, the upper one at the lower margin of the sternum and the lower one at the iliac crests. The organs and tissues that were exposed were the internal organs of the upper abdomen (liver, both adrenal glands, left kidney and upper part of the right kidney, colon, part of the stomach and some loops of the small intestine, part of the mesentery, etc.) and the lower parts of the lungs, in addition to the regional skin, subcutaneous tissue, muscular layers and peritoneal lining. The direction of the rays was antero-posterior, and 6 rats were irradiated at a time.

The standard conditions for irradiation were: Philips X-ray machine; physical factors: 200 kv, 18 m.a., FSD 50 cm., with 1·0 mm. Cu and 1·0 mm. Al filters; output 42 r/min. It should be pointed out, however, that somewhat different conditions were used for a relatively small number of animals, in groups 1 and 3, at the onset of the experiment (23 animals in each). The conditions of radiation were: Picker X-ray machine, 230 kv, 16 m.a., FSD 50 cm., 0·25 mm. Cu and 1·0 mm. Al filters; output 33 r/min. Furthermore, whereas the dose of partial-body radiation in the standard treatment was 700 r, in the early radiation series a dose of 1000 r was given. The fact that the mortality, in the early form of treatment, was rather high (9/23 in group 1 and 4/23 in group 3 during the first month) led to the change-over to the other treatment for the rest of the experiment. There was, however, no indication that the tumour distribution was significantly different among the survivors of those given the first form of treatment and those of the majority submitted to standard treatment.

The hepatectomies were performed under general anaesthesia, induced by nembutal (3·5 mg. per 100 g. of rat) and completed by ether inhalation, in small doses, to keep the animals quiet and relaxed during the operative procedure.

The hepatectomies were carried out by abdominal laparotomy—3 successive times at 10-day intervals. The first laparotomy wound was made along the outer margin of the left rectus muscle; the second, along the linea alba; and the third, along the outer margin of the right rectus muscle. Each laparotomy wound included sectioning of the skin and subcutaneous tissues, the rectus muscle and its fascia, and the corresponding peritoneal coat.

The partial hepatectomies involved, in the first operation, the amputation of the left portion of the medial lobe; in the second, removal of the right portion of the medial lobe; and in the third, removal of the right lateral lobe. The liver was each time sutured with silk, size 000, the resulting haemorrhage being slight.

The amounts of liver tissue resected (in grams) were as follows:

| | Maximum | Minimum | Average |
|---|---|---|---|
| At first operation | 2·8 | 0·9 | 1·6 |
| At second operation | 4·2 | 1·9 | 2·7 |
| At third operation | 4·4 | 0·6 | 2·0 |

The regeneration of the remaining portions of the liver could easily be observed at each successive operation, the amount of regeneration, as judged from a few preliminary tests, being each time approximately to the size of the original, normal liver.

The experiment was divided into 5 groups, according to the type of treatment received, as shown below, the figures in parenthesis giving the number of animals per group at the start of the experiment. (In view of the large numbers of animals used, the need for uniformity of age, and the limited numbers that could be irradiated or hepatectomized on any one day, the work was actually staggered over a period of several months, and the latent periods, etc., corrected at the end of the experiment.) All the animals were allowed to live their full life-span, except those that were moribund, which were sacrificed and examined. They were carefully autopsied after death, and all suspicious lesions, especially those indicating neoplastic changes, were fixed in Bouin and submitted to histological examination. The routine staining was with haematoxalin and eosin, but in special cases, iron haematoxalin, and occasionally other stains, were also used.

*Scheme of experiment*

*Group* 1 (124 rats): partial-body irradiation (standard, 700 r, with the exception of the first 23 animals, 9 of which died early, which received 1000 r); 7 days interval; then 3 partial hepatectomies at 10-days intervals.

*Group* 2 (126 rats): 3 partial hepatectomies at 10-day intervals, followed 7 days after the last operation, by standard 700 r X-irradiation.

*Group* 3 (115 rats): standard 700 r irradiation (with the exception of the first 23 animals which received 1000 r, 3 of which died early), with no hepatectomies or any other treatment before or after.

*Group* 4 (116 rats): 3 partial hepatectomies at 10-day intervals, with no other treatment.

*Group* 5 (110 rats): normal control rats of the same sex, age and weight.

### RESULTS

The tumours listed in Table I, comprising 84 from an effective total of 468 rats of the 5 groups (4 experimental and 1 untreated control), include a wide range of neoplasms affecting many different organs. In addition to those listed in Table I, 8 cases of benign fibroblastic lesions were also observed. Of the 84 tumours listed, 50 were sarcomas of various histological types; 26 were epithelial tumours of different types and sites of origin, of which 16 were definitely malignant, 7 were benign, and 3 others probably benign; and 8 were malignant lymphomas.

Of the 50 sarcomas, 49 appeared in the four experimental groups and only one in the untreated controls; and of these 49, 44 arose from tissues submitted to the experimental procedures—i.e. to irradiation and/or surgical operation (including the laparotomy wounds themselves). The remaining 5 sarcomas in the 4 experimental groups, which developed outside the treated areas, consisted of one tumour near the left eye, one arising from the upper part of the thoracic wall, two from the soft tissues of the leg, and one from the femur. (Of the 8 fibroblastic benign lesions, not listed in the table, 4 appeared within the treated zones and 4 at remote sites.)

All 26 tumours of epithelial origin developed within the treated zones—i.e. from the abdominal skin, liver, adrenal glands, kidneys, and intestine. Twenty-five of these were in the experimental groups 1–4 and only one in the untreated

TABLE I.—*Incidence of Tumours in Rats in response to X-irradiation and Hepatectomy*

| Groups | A Total tumours/effective total* | % | B Tumours in treated zone | % | C Total sarcomas | % | D Sarcomas in treated zone† | % | E Total epithelial tumours | % | F Epithelial tumours (excluding hepatomas) | % | G Hepatomas | % | H Malignant lymphomas | % |
|---|---|---|---|---|---|---|---|---|---|---|---|---|---|---|---|---|
| 1. X-rays followed by hepatectomy | 31/92 | 34 | 28 | 30 | 20 | 22 | 19 | 21 | 9 | 10 | 6 | 7 | 3 | 3 | 2 | 2 |
| 2. Hepatectomy followed by X-rays | 21/92 | 23 | 17 | 18 | 12 | 12 | 12 | 12 | 5 | 5 | 4 | 4 | 1 | 1 | 4 | 4 |
| 3. X-rays alone . . . | 26/96 | 27 | 22 | 23 | 15 | 16 | 12 | 12·5 | 10 | 10 | 8 | 8 | 2 | 2 | 1 | 1 |
| 4. Hepatectomy alone . . | 3/78 | 4 | 2 | 3 | 2 | 3 | 1 | 1 | 1 | 1 | 1 | 1 | 0 | 0 | 0 | 0 |
| 5. Untreated controls . . | 3/110 | 3 | 2‡ | 2 | 1 | 1 | 1‡ | 1 | 1 | 1 | 1 | 1 | 0 | 0 | 1 | 1 |
| Total . . . | 84/468 | | 71 | | 50 | | 45 | | 26 | | 20 | | 6 | | 8 | |

* Effective total = number of survivors at the time of appearance of the first tumour in the group.

† Including sarcomas of the liver.

‡ Since group 5 received no treatment, the figures for this group under columns B and D represent tumours in the region corresponding to the treated zones in the other groups.

controls. Six of these epithelial tumours arose from the liver parenchyma—3 probably benign and 3 malignant hepatomas.

As regards the incidence and distribution of the tumours in relation to the experimental treatment received by the animals of the various groups, only 3/110 (3 per cent) appeared in the untreated controls and 3/78 (4 per cent) in group 4, submitted to surgical procedures (laparotomies and partial hepatectomies) without radiation treatment before or after, in contrast to 31/92 (34 per cent) in group 1, receiving X-rays followed by repeated hepatectomies, 21/92 (23 per cent) in group 2, receiving the double treatment in reverse, i.e. repeated hepatectomies followed by X-rays, and 26/96 (27 per cent) in group 3, receiving X-rays alone.

The only significant difference in tumour incidence noted among the three groups that received radiation treatment (groups 1–3) was in connection with the sarcomas within the treated zones (see column D in Table I); and when the different types of sarcoma were examined separately, the difference was found to be confined to rhabdomyosarcomas (see Table II): Of the 9 rhabdomyosarcomas in the whole series, seven appeared in group 1, one each in groups 2 and 4, and none in groups 3 and 5.

Two other noteworthy features of these results were (a) the early appearance of the rhabdomyosarcomas in group 1, in striking contrast to the late appearance of most other tumours in all the groups, and (b) the high incidence of metastases among the rhabdomyosarcomas.

The following is a summary of the latent periods for the various categories:

*For rhabdomyosarcomas*, in group 1—mean latent period: 273 days; range: 238–343 days; in group 2—360 days (1 tumour only); in group 3—790 days (1 tumour only).

*For fibrosarcomas*, in group 1—mean latent period: 600 days; range: 410–779 days; in group 2—mean latent period: 536 days; range: 320–660 days.

For further comparison, the mean latent period for hepatomas was 560 days, and for adrenal tumours, 643 days.

The incidence of metastases among the different tumour types is shown in Table III. The distribution and character of the metastases will be discussed later.

*Histology*

In view of the unusual distribution of one particular type of sarcoma among the different experimental groups, special attention was paid to the histopathological features of all the sarcomas in the series.

*Fibrosarcomas.*—All the tumours classified as fibrosarcomas had the basic histopathological pattern of interlacing bundles of elongated cells and fibres arranged in parallel linear or curved distribution. Structural variations in cellularity, in cell size and in the amount of intercellular fibres, were often encountered in different parts of the same tumour. Following the histopathological criteria outlined by Stout (1948), two categories of fibrosarcomas could be distinguished according to the prevailing features in each tumour: poorly-differentiated and well-differentiated fibrosarcomas.

In the poorly-differentiated variant, the neoplastic cells were closely packed and showed some degree of anaplasia, with variations in size and frequent mitotic

TABLE II.—*Distribution of Various Sarcoma Types in Relation to Treatment Received*

| Groups | Fibro-sarcomas | Rhabdomyo-sarcomas | Other sarcomas | Total sarcomas |
|---|---|---|---|---|
| 1. *X-rays followed by hepatectomy :* | | | | |
| In body wall | 6 | 7 | 1 | 14 |
| In liver | 3 | 0 | 1 | 4 |
| Intra-abdominal | 2 | 0 | 0 | 2 |
| Outside treated zone | 0 | 0 | 0 | 0 |
| Total | 11 | 7 | 2 | 20 |
| 2. *Hepatectomy followed by X-rays :* | | | | |
| In body wall | 4 | 1 | 1 | 6 |
| In liver | 3 | 0 | 1 | 4 |
| Intra-abdominal | 1 | 0 | 1 | 2 |
| Outside treated zone | 0 | 0 | 0 | 0 |
| Total | 8 | 1 | 3 | 12 |
| 3. *X-rays alone :* | | | | |
| In body wall | 5 | 0 | 2 | 7 |
| In liver | 0 | 0 | 0 | 0 |
| Intra-abdominal | 3 | 0 | 1 | 4 |
| Outside treated zone | 2 | 0 | 2 | 4 |
| Total | 10 | 0 | 5 | 15 |
| 4. *Hepatectomy alone :* | | | | |
| In body wall | 0 | 1 | 0 | 1 |
| In liver | 0 | 0 | 0 | 0 |
| Intra-abdominal | 0 | 0 | 0 | 0 |
| Outside treated zone | 1 | 0 | 0 | 1 |
| Total | 1 | 1 | 0 | 2 |
| 5. *Untreated controls :* | | | | |
| In body wall | 0 | 0 | 0 | 0 |
| In liver | 0 | 0 | 0 | 0 |
| Intra-abdominal | 0 | 0 | 1 | 1 |
| Outside zone | 0 | 0 | 0 | 0 |
| Total | 0 | 0 | 1 | 1 |
| Total | 30 | 9 | 11 | 50 |

TABLE III.—*Incidence of Metastasis among the Malignant Tumours**

| Groups | Fibro-sarcoma | Rhabdo-myosarcoma | Other sarcomas | Hepatomas | Epithelial tumours (excluding hepatomas) | Total |
|---|---|---|---|---|---|---|
| 1. X-rays followed by hepatectomy | 2/11 | 6/7 | 0/2 | 1/3 | 1/6 | 10/29 |
| 2. Hepatectomy followed by X-rays | 1/8 | 1/1 | 0/3 | 0/1 | 0/4 | 2/17 |
| 3. X-rays alone | 0/10 | — | 1/5 | 0/2 | 0/8 | 1/25 |
| 4. Hepatectomy alone | 0/1 | 0/1 | — | — | 0/1 | 0/3 |
| 5. Untreated controls | — | — | 0/1 | — | 0/1 | 0/2 |
| Total | 3/30 | 7/9 | 1/11 | 1/6 | 1/20 | 13/76 |

* Malignant lymphomas not included.

figures, some of the latter of bizarre shape. The fibrillar intercellular component was scarce and consisted of delicate reticulin fibrils.

In the well-differentiated fibrosarcomas, the tumour tissue showed less cellularity, with well developed collagen fibres and elongated spindle-cells of relatively uniform size. Very few mitoses were seen in this type of sarcoma. Examples of intermediate types between the two histological variants were also encountered (Fig. 1). A few of the fibrosarcomas arising from the subcutaneous tissues displayed an architectural pattern closely resembling that described in human pathology as " dermatofibrosarcoma ", with a characteristic interwoven arrangement of neoplastic cells and fibres.

At the periphery of the neoplastic growth, the tumours seemed, in some cases, to be separated from the adjacent tissues by a capsule-like structure, probably resulting from compression of the neoplastic tissue itself. More often, the proliferating cells infiltrated diffusely the neighbouring muscle and fat. The invaded muscle bundles were widely separated, with single muscle fibres sometimes isolated and completely embedded in the tumour tissue. Among the neoplastic cells, some scattered large syncytium-like cellular formations were found which had the morphological character of the so-called " muscle giant-cell ". These cells could easily be distinguished from the atypical monster giant-cells of the rhabdomyosarcomas, and were therefore interpreted as representing regenerating buddings arising from injured pre-existing muscle fibres, as distinct from neoplastic muscle giant-cells.

In some of the fibrosarcomas of groups 1 and 2, areas of scar tissue were found included in the tumour mass. At the margin of such areas, the fibrous tissue of the scar merged gradually with the surrounding neoplastic tissue. There was not sufficient histological evidence, however, to enable one to decide whether the sarcomatous growth originated from the pre-existing connective tissue of the abdominal wall and liver, respectively, or from the connective tissue of the scar which developed as the consequence of the surgical operation.

In those cases in which both the liver and the abdominal wall were extensively infiltrated by the fibrosarcoma, it sometimes proved difficult to determine, on a mere histological basis, the primary site of origin of the tumour. This had, in such cases, to be inferred from gross criteria.

*Rhabdomyosarcomas.*—The essential histopathological features of the tumours that were diagnosed as rhabdomyosarcomas were great cellularity and an extreme degree of cellular pleomorphism. The prevailing elements included spindle or oval cells of varying size, together with elongated strap-like or ribbon-shaped cells, and monster giant-cells of extreme bizarre shape (Fig. 2).

The giant-cells contained several nuclei arranged more or less in linear series or as hyperchromatic clumps, often located at one or both ends of the cellular body (Fig. 3). Cells of " racquet " or " tadpole " shape with tails varying in length were frequently encountered. The abundant cytoplasm of the larger cells stained intensely with eosin, and often displayed a homogeneous or finely granular fibrillar appearance. No distinct cross-striation could be detected, in spite of careful search under high magnification and special staining techniques. Nevertheless, the other histological features were considered to furnish adequate evidence for establishing the rhabdomyoblastic nature of these tumours.*

* In view of the element of uncertainty sometimes felt in reaching a diagnosis of rhabdomyosarcoma in the absence of evidence of cross-striations, the sections were shown to a number of visiting pathologists, and our diagnosis confirmed in every case.

In two cases, a somewhat different pattern prevailed, the usual pleomorphic picture being confined to small, circumscribed areas, the pattern repeating itself in most of the sections of the particular tumours. The tumour, in both instances, consisted mainly of round or oval cells of moderate size, relatively unattached to one another. These cells were contained in large irregular spaces limited by thin stromal trabeculae. The cells showed no marked variability in size, although large multinucleated cells were occasionally seen. The cytoplasm was strongly eosinophilic and finely granular, and many atypical mitotic figures were also present. Characteristically, in the alveolar spaces, the cells close to the septa appeared to be attached to the latter by a narrow pedicle, the nucleus being located at the opposite, bulbous end of the cell (Fig. 4). The whole pattern had, therefore, a striking resemblance to the histological type of rhabdomyosarcoma recently described by Riopelle and Thériault (1956) and designated by them as " alveolar rhabdomyosarcoma ", as a morphological variant of the more common pleomorphic type.

In one case, a few irregularly arranged trabeculae of newly-formed bone were found within the tumour, which otherwise had the characteristic feature of a rhabdomyosarcoma. The same finding has previously been reported in spontaneous rhabdomyosarcoma of the mouse (Nettleship, 1943 ; Hurley, 1956). As in the case reported by Hurley, no evidence of transition between the neoplastic tissue and the bone could be found. It was, therefore, assumed that the osseous trabeculae originated as a metaplastic change in the stromal tissue.

In seven of the nine cases of rhabdomyosarcoma, metastases were found in distant organs (lungs, lymph nodes, peritoneum, etc.), in contrast to the relatively rare occurrence of metastases with the other types of sarcoma or carcinoma in the present series (Table III). The tumour cells in the metastatic foci retained most of the features of the primary growths, including the capacity for muscle differentiation (Fig. 5). Many multinucleated giant-cells with characteristic strap-like appearance were found in all the metastatic nodules.

*Other types of sarcoma.*—The following types of sarcoma were also encountered, all of them confined to the treated zones unless otherwise stated :—One leiomyosarcoma and one undifferentiated spindle-cell sarcoma, in group 1 ; two leiomyosarcomas and one undifferentiated sarcoma, in group 2 ; one chondrosarcoma, one leiomyosarcoma and one undifferentiated sarcoma—all 3 in the treated zone—

---

EXPLANATION OF PLATES

FIG. 1.—Typical pattern of interlacing bundles in a partially-differentiated fibrosarcoma (intermediate type). H. and E. ×45.

FIG. 2.—Rhabdomyosarcoma. Cellular pleomorphism with many multinucleated giant cells of varying size and ribbon-shaped cells. H. and E. ×45.

FIG. 3.—Characteristic multinucleated giant cells, with spindle cells, in a pleomorphic rhabdomyosarcoma infiltrating subcutaneous tissue. H. and E. ×100.

FIG. 4.—Rhabdomyosarcoma of alveolar type composed of round or oval atypical cells. Note at the top some cells attached to a thin stromal trabecula. H. and E. ×200.

FIG. 5.—Metastasis of rhabdomyosarcoma in the lung showing multinucleated giant cells. H. and E. ×45.

FIG. 6.—Trabecular hepatoma with typical liver-cell architecture. H. and E. ×45.

FIG. 7.—Anaplastic carcinoma of the liver. Atypical large cells arranged in irregular cords. Mitotic figures are numerous. H. and E. ×100.

FIG. 8.—Adenocarcinoma of the liver. The glandular structures are lined with cuboidal epithelium. H. and E. ×45.

1

2

3

4

Haran-Ghera, Trainin, Fiore-Donati and Berenblum.

5 6

7 8

as well as one liposarcoma in the upper part of the thoracic wall and one osteosarcoma of the femur, in group 3 ; none in group 4 ; and one undifferentiated sarcoma in group 5.

*Malignant lymphomas.*—Eight malignant lymphomas were encountered. Of these, 6 were generalized lymphomas and 2 were localized in the mediastinal region. Their distribution according to groups is shown in Table I. With the exception of one case of myeloid leukaemia, all were of lymphoid type.

*Epithelial tumours.*—In addition to the sarcomas of the liver mentioned above 6 tumours of epithelial type were found arising in the liver. Three of these were in group 1 ; one in group 2 ; and two in group 3 (Table I).

Three of these liver tumours were solid hepatomas of liver-cell type, showing a trabecular patten with alternate blood sinusoids, mimicking the architecture of the normal liver tissue (Fig. 6). One of these was of microscopic size, discovered incidentally in the liver of a rat bearing a fibrosarcoma ; the other two were multicentric. In one of these, the tumour was composed mainly of multilayered sheets of small cells and included areas in which transition to gland-like structures was occasionally noted. Mitoses were rare or absent. The other tumour conconsisted of large cells arranged in cords of 2–3 cells thickness. The conservative diagnosis of " benign hepatoma " was made for these 3 tumours, though such innocent looking lesions have been known to metastasize.

The other 3 of the 6 epithelial tumours of the liver showed definite evidence of histological malignancy. One was an anaplastic carcinoma (Fig. 7), consisting of irregular trabeculae or islands of large, atypical cells, exhibiting remarkable variability in size and shape, and only rarely bearing any resemblance to normal hepatic cells. Many atypical mitotic figures were present. The remaining two tumours were composed of glandular structures of acinar or tubular type (Fig. 8), occasionally showing papillary formation and cystic dilatations of the glandular lumen. The glands were lined by single or multiple cells of cuboidal or columnar epithelium, showing a certain variability in size and shape. Nuclei were hyperchromatic, but rarely showing mitotic activity. No evidence of mucus secretion could be observed in any of the sections examined. One of these adenocarcinomas appeared to be multicentric in origin, with metastases in the peritoneum and lymph nodes.

*Other epithelial tumours.*—Eight adrenal cortical tumours, 5 benign and 3 malignant, were found in the present series. Their distribution, according to treatment, was as follows : two adenomas in group 1 ; one adenoma and three carcinomas in group 3 ; one adenoma in group 4 ; and one adenoma in group 5.

The remaining epithelial tumours comprised 4 renal carcinomas—one in group 1 ; one in group 2 ; and two in group 3 ; also 3 intestinal carcinomas—one each in groups 1, 2 and 3 ; two adenomas of the pancreas—one in group 1 and one in group 2 ; and 4 basal cell carcinomas arising from the skin within the treated zones—one in group 2 and three in group 3.

### DISCUSSION

The choice of X-rays, to serve as initiating stimulus for liver carcinogenesis in the present experiment, was made on the basis of previous reports (Koletsky and Gustafson, 1955 ; Upton, Kimball, Furth, Christenberry and Benedict, 1960) of borderline carcinogenicity for the liver by whole-body radiation. The fact that other investigators (Finerty, Binhammer, Schneider and Cunningham, 1953), reporting on a wide spectrum of X-ray-induced tumours, did not observe liver tumours in their series, confirmed the " borderline " nature of the action of X-rays on the liver, and made radiation appear all the more promising as a " pure " initiator for that organ. In the present experiment, in which the radiation was not whole-body but confined to the region of the liver, 2/96 hepatomas appeared in group 3, which received X-ray treatment alone.

The technique of successive partial hepatectomies, as a means of prolonging the resulting reparative hyperplasia, for promoting action, was made possible by the fact that the rat liver is multi-lobulated. Preliminary tests showed that considerable regeneration occurred after each successive operation, the weight of the liver increasing every time back to that of the normal liver.

While the experimental conditions chosen thus appeared, on *a priori* grounds, to have been satisfactory and critical, and while adequate numbers of animals were used (more than 100 rats per group), the result of the experiment, with respect to the question of a two-stage mechanism being operative in liver carcinogenesis, was essentially negative: Only 3 hepatomas appeared in group 1 (radiation followed by hepatectomy); 1 hepatoma in group 2 (hepatectomy followed by radiation); and 2 hepatomas in group 3 (radiation alone).

One cannot necessarily conclude, because the methods used here were more exacting than those used by Glinos *et al.* (1951), that our negative results outweigh or contradict their positive results. All that can be deduced is that exposure of the liver to X-rays does not seem to function as an effective stimulus for initiating action for that organ.

The alternative possibility—that the fault lay in the use of reparative regeneration as the intended promoting stimulus—is rendered unlikely (a) by the results of Glinos *et al.* themselves; (b) by the recent observation of Rosen and Cole (1962) that the development of tumours of the kidney in mice, in response to X-radiation, is augmented (in the remaining kidney) by unilateral nephrectomy; and (c) by our own results, from the present experiment, of the development of rhabdomyosarcomas at the site of repair in laparotomy wounds in previously irradiated animals.

Spontaneous rhabdomyosarcomas are very rare in rodents (Nettleship, 1943) and other animal species (Worley and Gorham, 1954), though they have been frequently reported to occur among the carcinogen-induced sarcomas in mice and rats (Haagensen and Krehbiel, 1936; Burdette and Strong, 1943; Saxén, 1953).

The rarity of cross-striation in histological sections of such tumours need not prevent one from making the diagnosis of rhabdomyosarcoma, according to Stewart (1953), provided the other characteristic histological features are present. Bonser and Orr (1939), however, expressed the view that the muscle giant-cells in the majority of pleomorphic sarcomas induced by carcinogenic hydrocarbons represent a reaction to injury of pre-existing muscle fibres, and that such tumours should not, therefore, be classed as being derived from muscle cells. Such changes undoubtedly occur in many of the induced sarcomas, and have been noted in the sarcomas of the present series. This interpretation could, however, be ruled out in the tumours of the present experiment classified as rhabdomyosarcomas, since the same pleomorphic pattern *with characteristic muscle giant-cells* was found in all the metastatic foci, i.e. in the lungs, liver and lymph nodes, where pre-existing striped muscles could not be expected to have been present.

The high incidence of rhabdomyosarcomas in the abdominal scars of group 1 (X-rays followed by hepatectomy) and the very low incidence in group 2 (hepatectomy followed by X-rays), suggests the possibility of a two-stage mechanism, whereby the radiation served as initiating factor and the wound repair resulting from the laparotomies served as promoting factor. The fibrosarcomas, on the other hand, seemed to be uniformly distributed among the various groups of irradiated animals—i.e. irrespective as to whether the radiation treatment was preceded or followed by wound healing, or was not accompanied by wound healing at all.

Ionizing radiation would thus appear to be rather selective for the different cell types in its capacity to act as an initiating stimulus for carcinogenesis ; though the nature of the subsequent promoting stimulus is also an important factor. Ross, Keep and Moritz (1959), for instance, failed to augment the carcinogenic action of whole-body radiation on the skin of rats by subsequent thermal burns. On the other hand, there are the positive effects of nephrectomy, by Rosen and Cole (1962) quoted above, as well as the evidence of radiation acting as the initiating stimulus for leukaemogenesis, with urethane acting as promoting agent (Berenblum and Trainin, 1960). Mention should also be made, in this connection, of the observation by Shubik, Goldfarb, Ritchie and Lisco (1953), that beta-radiation could act as an initiating stimulus for skin carcinogenesis, with croton oil painting serving as the promoting factor.

### SUMMARY

In a study on the influence of partial-body X-irradiation of the liver and surrounding tissues, preceded or followed by repeated partial hepatectomies, no evidence was found to support the notion that liver regeneration, resulting from repeated hepatectomies, could serve as a promoting stimulus, with radiation serving as initiating stimulus, for liver carcinogenesis in the rat.

Many tumours of diverse types did, however, develop in the irradiated animals ; and of these, a high proportion were sarcomas—i.e. 20 out of a total of 31 tumours in the group receiving X-rays followed by hepatectomy, 12 out of a total of 21 tumours in the group receiving hepatectomy followed by X-rays, and 15 out of a total of 26 tumours in the group with X-rays alone ; while 2 out of 3 were in the group receiving hepatectomy alone and 1 out of 3 in the untreated control group.

Of the tumours within the treated zone only, the numbers of sarcomas were 19 with X-rays followed by hepatectomy, 12 with hepatectomy followed by X-rays, and 12 with X-rays alone.

When the data were analysed in terms of the separate histological types, it was observed that of the 9 cases of rhabdomyosarcoma, 7 were in the abdominal scars in the group receiving X-rays followed by hepatectomy, but only 1 in the group in which the two treatments were reversed, and none in the group receiving X-rays alone. The incidence of X-ray-induced fibrosarcomas, on the other hand, was not apparently influenced by wound healing, whether before or after the irradiation.

The results suggest the possibility of a two-stage mechanism of carcinogenesis with respect to rhabdomyosarcoma induction, whereby X-irradiation acted as initating factor and wound healing (following laparotomy) acted as promoting factor.

This work was supported in part by a grant from the Joseph and Helen Yeamans Levy Foundation, U.S.A., to whom the authors wish to express their indebtedness.

### REFERENCES

Berenblum, I. and Trainin, N.—(1960) *Science*, **132**, 40.
Bonser, G. M. and Orr, J. W.—(1939) *J. Path. Bact.*, **49**, 171.
Burdette, W. J. and Strong, L. C.—(1943) *Cancer Res.*, **3**, 13.
Finerty, J. C., Binhammer, R. T., Schneider, M. and Cunningham, A. W. B.—(1953) *J. nat. Cancer Inst.*, **14**, 149.
Glinos, A. D., Bucher, N. L. R. and Aub, J. C.—(1951) *J. exp. Med.*, **93**, 313.
Haagensen, C. D. and Krehbiel, O. F.—(1936) *Amer. J. Cancer*, **26**, 368.
Hurley, J. V.—(1956) *J. Path. Bact.*, **72**, 690.
Koletsky, S. and Gustafson, G. E.—(1955) *Cancer Res.*, **15**, 100.
Nettleship, A.—(1943) *J. nat. Cancer Inst.*, **3**, 563.
Riopelle, J. L. and Thériault, J. P.—(1956) *Ann. d'anat. path.*, **1**, 88.
Rosen, V. J. and Cole, L. J. (1962) *J. nat. Cancer Inst.*, **28**, 1031.
Ross, O. A., Keep, P. and Moritz, A. R.—(1959) *Arch. Path.*, **67**, 103.
Saxén, E. A.—(1953) *J. nat. Cancer Inst.*, **14**, 547.
Shubik, P., Goldfarb, A. R., Ritchie, A. C. and Lisco, H.—(1953) *Nature, Lond.*, 171, **934**.
Stewart, H. L.—(1953) in ' The Physiopathology of Cancer '. New York (Hoeber-Harper), p. 46.
Stout, A. P.—(1948) *Cancer*, **1**, 30.
Upton, A. C., Kimball, A. W., Furth, J., Christenberry, K. W. and Benedict, W. H.—(1960) *Cancer Res.*, **20** (No. 8, Part 2), 1.
Worley, G. and Gorham, J. R.—(1954) *Amer. J. Path.*, **30**, 837.